# Beyond the apnea-hypopnea index: symptomatic assessment as a treatment pathway for obstructive sleep apnea management

**DOI:** 10.1093/sleep/zsaf111

**Published:** 2025-05-30

**Authors:** Fernanda R Almeida, Susana Falardo

**Affiliations:** Division of Orthodontics and Dental Sleep Medicine, Department of Oral Health Sciences, Faculty of Dentistry, University of British Columbia, Vancouver, Canada; Postgraduate Sleep Medicine Course, Catholic Medical School, Universidade Católica Portuguesa, Lisbon, Portugal

Obstructive sleep apnea (OSA) is a highly prevalent condition, with positive airway pressure (PAP) being the most studied and widely used treatment modality. While a variety of non-PAP therapies have been explored over the years, none have demonstrated the same efficacy in addressing the apnea-hypopnea index (AHI) as PAP therapy. PAP is capable of almost completely eliminating breathing events, regardless of the patient’s age, body mass index, endotypes, or comorbidities. However, there is a critical issue to address: AHI has not been consistently correlated with symptomatic outcomes, such as daytime sleepiness and overall sleep quality. By analyzing clinical subtypes and their symptomatic profiles, various studies have proposed that a broader approach could improve diagnostic accuracy and better predict cardiovascular outcomes [1, 2].

Questions remain regarding what constitutes an excessive number of apneas and hypopneas and to what extent these parameters are associated with adverse health outcomes. Over the past three decades, the rapid advancement of medical technologies has led to significant changes in how hypopneas are defined and diagnosed. For instance, the use of nasal cannulas to detect reductions in airflow, combined with electroencephalography to correlate respiratory events with arousals, has contributed to an increased diagnosis of sleep apnea. The *HypnoLaus* study [3] and others [4] demonstrated that, based on current diagnostic criteria, 84% of men and 61% of women in the general population would be classified as positive for OSA. However, this study found that only an AHI greater than 20 events per hour was associated with comorbidities such as hypertension, diabetes, metabolic syndrome, and depression. The American Thoracic Society Task Force [5] reported limited benefits of treatment for mild OSA (AHI between 5 and 15 events per hour) and upper airway resistance. Despite this, there exists a subgroup of mild OSA patients who are highly symptomatic and experience significant improvements in daytime sleepiness and fatigue when treated. Unfortunately, because symptomatic assessment is not often used as a treatment criterion, many patients do not receive treatment, or treatments are not reimbursed.

It is important to recognize that the symptoms in many mild OSA patients are often a consequence of something other than OSA itself, such as sleep deprivation, environmental factors, or comorbid conditions like anemia, chronic bowel syndrome, or cancer [6]. In these cases, the use of PAP or other OSA therapies would not lead to symptomatic improvement, and the underlying condition remains undiagnosed. This phenomenon of overdiagnosis, has also been recognized in other diseases such as diabetes, cancer, and HIV [7], and may result in unnecessary treatments that do not benefit the patient.

While there is consensus regarding the treatment of moderate to severe OSA, the decision to treat mild OSA is less certain. Despite this knowledge, an AHI of less than 5 events per hour, minimal hypoxic burden, and other polysomnographic variables are still considered the benchmarks of therapeutic success, this fails to account for symptomatic improvement and lack of treatment need for mild OSA. Moreover, with therapies such as oral appliances (OAs), positional therapy, and surgery, which often reduce AHI to below 20 events per hour, patients are discouraged to continue treatment, regardless of symptomatic improvement.

OAs are recognized as an alternative to PAP, with a significant body of literature supporting their efficacy. Eleven randomized controlled trials (RCTs) and six well-conducted studies have shown that OAs can improve cardiovascular morbidity, as evidenced by reductions in 24-hour blood pressure and other cardiovascular proxies [8]. Moreover, 25 RCTs have demonstrated substantial improvements in quality of life for OSA patients using OAs, likely due to improvements in OSA severity and higher treatment adherence [9]. While OAs are generally less effective in controlling AHI and other polysomnographic parameters, their effectiveness in improving symptoms and blood pressure is comparable to that of PAP.

The *Crescent* trial, “Patient-Reported Quality of Life Outcomes with Mandibular Advancement versus CPAP: Insights from the CRESCENT Trial” [10], is an impressive long-term parallel study comparing the long-term effects of OAs versus PAP in patients with moderate to severe OSA and cardiovascular comorbidities. The trial enrolled a cohort with an average age of 61 years, including 44% overweight and 49% obese patients, 61% at high risk for coronary artery disease, 60% high risk for diabetes and all patients had known hypertension with 44% with high blood pressure for at least 10 years. Previous studies comparing PAP to OA [11, 12] have hypothesized that similar symptomatic improvement in OA was related to higher adherence, but the crescent trial could not find a strong correlation between adherence and quality of life. In this study, OA reduced the AHI from 37 to 11/h with a mean adherence of 5.5 hours, whereas PAP reduced from 39 to 2/h with a mean adherence of 5.5 hours. In a previous publication, the Crescent trial showed a non-inferiority of OA to PAP in the reduction of 24-hour blood pressure [13]. Despite these differences, both treatments improved effectively the quality of life, sleep-related quality of life, and sleepiness, with PAP being superior in the improvement of sleep-related quality of life (QoL). This highlights the move to precision medicine, where the severity of the disease should include symptoms such as anxiety, depression, measures of attention, sleepiness, and quality of life and biological activity such as blood pressure, C-reactive protein, and HbA1c levels [14].

In accordance with the American Academy of Sleep Medicine’s 2015 position paper [15] and the 2021 European Respiratory Society guidelines [16], OA which includes only the mandibular advancement devices are considered a viable treatment option for patients with mild to severe OSA who cannot tolerate PAP or prefer OA therapy. However, OA are not recommended for patients with severe OSA (AHI > 30 events per hour) with comorbidities like hypertension. Given that PAP adherence remains suboptimal, many patients remain untreated following diagnosis or initial PAP trials. The *Crescent* trial challenges the conventional treatment approach and may prompt a reevaluation of current guidelines, emphasizing the need for personalized treatment strategies in OSA.

Patient-centered care is crucial in the management of chronic conditions such as OSA. Understanding patient preferences can enhance treatment adherence and overall care effectiveness [17]. Patient-centered medicine incorporates treatment goals defined by the patient, taking into account their values, prior experiences, acceptability of treatment, and awareness of both the disease and available therapies [15]. Discrete choice experiments and decision aids [18, 19] have shown that patients prioritize improving their health, reducing apnea, enhancing sleep quality, and alleviating daytime sleepiness, while balancing factors such as comfort, side effects, cost, and convenience. Treatment satisfaction, while an important consideration, is often not explicitly addressed as a primary treatment goal.

In summary, the *Crescent* trial highlights the need to rethink current treatment strategies for OSA. Based on an excellent research methodology, it illustrated that oral appliances may offer a viable treatment alternative for patients with severe OSA and cardiovascular comorbidities, demonstrating comparable effectiveness to PAP therapy. Future research should prioritize patient preferences and treatment satisfaction as key outcomes, as well as symptomatic and biologic activity improvements [14] and focus on developing personalized treatment approaches that consider both clinical and symptomatic factors in the management of OSA to decide which is the ideal path to improve overall OSA management.
